# Network-based gene prediction for *Plasmodium falciparum *malaria towards genetics-based drug discovery

**DOI:** 10.1186/1471-2164-16-S7-S9

**Published:** 2015-06-11

**Authors:** Yang Chen, Rong Xu

**Affiliations:** 1 Department of Epidemiology and Biostatistics, Case Western Reserve University, Cleveland, OH, USA; 2 Department of Electrical Engineering and Computer Science, Case Western Reserve University, Cleveland, OH, USA

**Keywords:** malaria, disease gene prediction, network analysis, drug discovery

## Abstract

**Background:**

Malaria is the most deadly parasitic infectious disease. Existing drug treatments have limited efficacy in malaria elimination, and the complex pathogenesis of the disease is not fully understood. Detecting novel malaria-associated genes not only contributes in revealing the disease pathogenesis, but also facilitates discovering new targets for anti-malaria drugs.

**Methods:**

In this study, we developed a network-based approach to predict malaria-associated genes. We constructed a cross-species network to integrate human-human, parasite-parasite and human-parasite protein interactions. Then we extended the random walk algorithm on this network, and used known malaria genes as the seeds to find novel candidate genes for malaria.

**Results:**

We validated our algorithms using 77 known malaria genes: 14 human genes and 63 parasite genes were ranked averagely within top 2% and top 4%, respectively among human and parasite genomes. We also evaluated our method for predicting novel malaria genes using a set of 27 genes with literature supporting evidence. Our approach ranked 12 genes within top 1% and 24 genes within top 5%. In addition, we demonstrated that top-ranked candied genes were enriched for drug targets, and identified commonalities underlying top-ranked malaria genes through pathway analysis. In summary, the candidate malaria-associated genes predicted by our data-driven approach have the potential to guide genetics-based anti-malaria drug discovery.

## Background

Malaria is the most deadly parasitic infectious disease, which killed 627,000 people and caused 482,000 childhood deaths worldwide in 2012 [1]. Existing drug treatments show limited efficacy in malaria elimination [2-6]. Detecting the novel genetic basis for malaria not only reveals the disease pathogenesis, but also facilitates discovering new targets for anti-malaria drugs [7-11].

The pathogen causing malaria is the *Plasmodium *species. After being injected by mosquitos into human skin, these parasites infect the liver and multiply using the host's cell resources. Then they invade the red blood cells and cause the disease symptoms [12-14]. In both the liver and blood stage, the parasites trigger the host's innate immune responses and remodel the host cells to survive from the immune responses [15-19]. The complex pathogenesis of malaria involves both human and parasite genomes [20-24], and is not fully understood yet [25-27].

Studies of the human-parasite protein interactions have provided insights into the molecular signatures for malaria-specific host immune responses [20,28,29]. For example, studies show that the parasite protein PfEMP1 binds the human protein CD36 [30-32] and ICAM1 [33-35], which play critical roles in the adhesion of the infected red blood cells to the endothelial cells, and eventually lead to the disruption of bloodbrain barrier in cerebral malaria patients [36,37]. Another example shows that the PfRh family of proteins in the parasites directly interacting with the human protein CR1 during the invasion of red blood cells, and CR1 has the potential to become the target of blood-stage vaccines [38,39].

Currently, large-scale data have been accumulated on the human genome, parasite genome and their interactions. Integration and systematic analysis of the cross-species genomic data may lead to novel discoveries in genetic basis of malaria. In this study, we designed a data-driven approach to infer novel malariaassociated genes. Recent computational disease gene discovery algorithms have shown great potential in predicting disease causes [40-48]. They exploited the protein interactome in human genome and assumed that genes related to a disease phenotype tend to be located in a neighborhood in the protein-protein interaction network [49]. However, traditional methods are not sufficient for predicting genes for malaria, which naturally involves human-parasite protein interactions. Our approach represented the interacting human and parasite genomes with a heterogeneous network. We prioritized genes that are functionally related to the known malaria genes in the heterogeneous network and investigated if the top-ranked genes have the potential to guide drug discovery for malaria. We made our results publicly accessible at http://nlp.case.edu/public/data/malaria.

## Methods

**Figure 1:**
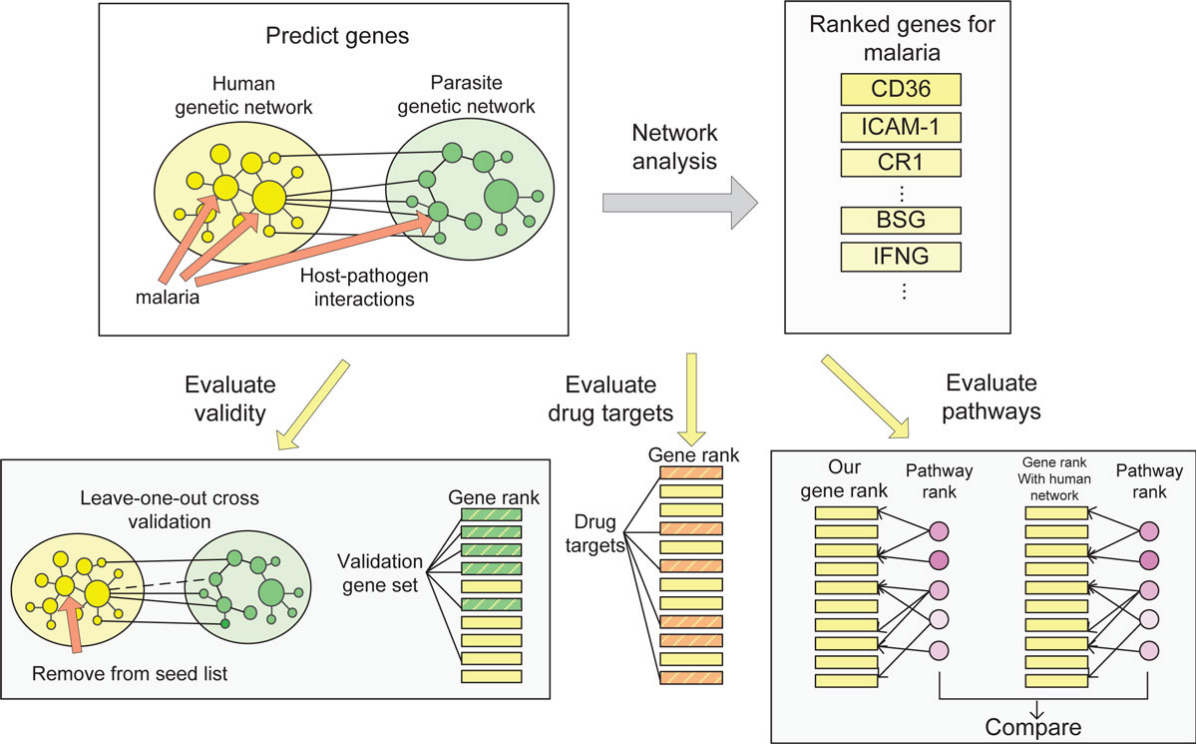
**Our methods contain two parts: gene prediction and result analysis**. We predicted genes associated with malaria from the cross-species genetic networks. We also evaluated the method validity in predicting malaria genes, distribution of druggable genes among the rank, and the pathways associated with the top-ranked genes.

Our experiment work flow is depicted in Figure 1 and consists of two steps: (1) prioritize genes through network analysis and (2) analyze the result. We first constructed separate genetic networks for human genome and parasite genome, and then connected them with host-pathogen protein interactions. We used genes that are known to be associated with malaria as the seeds and applied a random-walk based algorithm to rank genes in the cross-species network. To validate our method in prioritizing malaria genes, we performed a "leave-one-out" cross validation analysis and examined the ranks of a set of malaria genes extracted from literature. Then we evaluated if the top-ranked genes are druggable. Finally, we analyzed the functions of the prioritized genes by extracting pathways on the basis of gene ranking.

### Construct cross-species gene network

We constructed the genetic network for human and *Plasmodium falciparum *(the species that causes the most dangerous form of malaria) from the STRING [50,51] database. STRING includes gene relationships over a thousand species from four sources:protein-protein interactions (PPIs) databases, PPIs mined from literature abstracts, curated pathway databases and co-expressed genes.We used the four sources to build comprehensive networks for both human and *Plasmodium falciparum*.The human network contains 20,770 proteins and 4,850,628 interactions; and the *Plasmodium falciparum *network contains 4,913 proteins and 1,007,938 interactions. In addition, we used the scores from STRING to weight the edges in the two genetic networks.

We connected these two protein networks with 36 interactions from PathogenPortal [52] and literature [29,30,33]. These interactions are binary and cover physical associations, direct interactions and chemical reactions between the two species. The interaction pairs from literature were curated manually. We unified the gene identifiers with the genetic networks for human and parasites through HUGO Gene Nomenclature Committee [53] and PlasmoDB [54].

### Predict candidate genes for malaria

We manually collected 77 known malaria genes and used them as the seeds in our algorithm to find additional malaria genes. Among the 77 seed genes, 14 human genes were extracted from Online Mendelian Inheritance in Man (OMIM). In addition, extensive literature evidence suggests that the *Plasmodium falciparum *proteins--PfEMP1 [55-57], PfRh4 [38,58,59] and PfRh5 [60-62]-- are essential for parasite growth and red blood cell invasion. We extracted 63 parasite genes encoding these three proteins and added them into the seed list.

We initiated a random walk on the cross-species genetic network from the seeds, and ranked all the genes by the probabilities of being reached from the seeds. We extended the algorithm by regulating the movements of the random walker between networks with the jumping probabilities *λ*. We represented the human and parasite genetic network with *H *and *P *, respectively. When the random walker stands on a node in *H*, which is connected with a node in *P *, it may jump to *P *with the probability *λ *or stay in *H *with the probability of 1 − *λ*.

We calculated the ranking scores for each node as follows. Assume *p*0 is a vector of initial scores for each node, *p_k _*is the score vector at step k and was iteratively updated by:

(1) $$p_{k+1}=(1-\gamma )M^{T}p_{k}+\gamma p_{0}$$

where *γ *is the probability that the random walker restarts from the seeds at each step, and *M *is the transition matrix of the cross-species genetic network:

(2) $$M=\left[\begin{array}{cc} M_{H} & M_{HP}\\ M_{HP^{T}} & M_{P} \end{array}\right]\;.$$

The diagonal sub-matrices *M_H _*and *M_P _*consist of intra-network transition probabilities and were calculated as:

(3) $${{\left(M_{i}\right)}_{k}}_{l}=\left(\text{1}-\lambda x\right){\left(A_{i}\right)}_{kl}/\underset{l}{\sum }{\left(A_{i}\right)}_{kl},$$

where *i *∈ {*H, P *}, *A_i _*is the adjacency matrix of the network *H *or *P , k *is the index of row, *l *is the index of column, and *x *is an indicator variable, which equals to 1 if $\underset{l}{\sum }{\left(A_{i}\right)}_{kl}\neq 0$ and 0 otherwise. The off-diagonal sub-matrices *MHP *and *MHP T *consist of inter-network transition probabilities and were calculated as:

(4) $${\left(M_{j}\right)}_{kl}=\lambda x{\left(A_{j}\right)}_{kl}/\underset{l}{\sum }{\left(A_{j}\right)}_{kl},$$

where *j *∈ {*HP, HP ^T ^*} and × is the same indicator variable. While the method could obtain a score for each human and parasite gene, we focus on ranking and analyzing the human genes in this study.

### Evaluate the validity in predicting malaria genes

Before we used our method to predict genes for malaria, we performed the "leave-one-out" cross validation analysis to validate the method. Each time, we left out one malaria gene from the seed list, used the rest seeds as the input, and examined the rank of the excluded seed among the genes from the human or parasite genome. We repeated the same procedure for each of the 77 seeds, and assumed that the excluded seeds can be ranked highly if the method works well.

Then we used all the 77 seeds as the input, and evaluated if our gene ranking can prioritize novel malaria genes (other than the seeds). We manually constructed an independent set of 27 human genes involving malaria resistance and the host immune responses triggered by malaria parasites. These genes were extracted from literature references, which were mentioned in the textual descriptions of malaria in OMIM, and have zero overlap with the seed genes. We used this set as a proxy of novel malaria genes and evaluated the rank of this gene set among all human genes.

### Evaluate the ranks of druggable genes

Currently, only a subset of the human genome is druggable [63]. In this study, we investigated if the topranked genes represent opportunities for drug discovery for malaria. We first extracted 1,935 human genes that were targets of all drugs from DrugBank [64]. All these drug target genes appear in our genetic network and have no overlap with the seeds. We used all 77 seeds as the input and ranked the human genes. Then we calculated the number of target genes among every 500 human genes in the rank from the top to the bottom, and plotted the variation of this number.

### Extract and analyze malaria-specific pathways based on gene ranking

To better understand the functions of the prioritized genes, we linked the top 10% of human genes to their pathways. We downloaded 1320 canonical pathways from MSigDB [65] and ranked them based on the average of random walk scores for all the genes in each pathway. We manually examined if the top pathways are associated with the host response to the pathogen invasion.

In addition, we evaluated the impact of introducing the parasite genome into our gene prediction method. We removed the parasite genetic network and hostparasite interactions from our method, and calculated the random walk scores for human genes. Then we re-ranked the pathways containing the top 10% genes again. We compared the rank of pathways before and after using the parasite genetic network, and extracted the ones with largest rank difference.

## Result

### Network-based approach allowed the prioritization of known malaria genes from both human and parasite genomes

Among the 77 seed genes, 14 were human genes and 63 were parasite genes. We evaluated the performances of our algorithms in ranking human and parasite seed genes separately with a leave-one-out cross validation analysis. Our method required two parameters: the jumping probability *λ *between human and parasite genetic networks and the probability *γ *that the random walker restarts from the seeds. We chose *λ*=0.8 and *γ*=0.3 to achieve the best performance in the cross validation, but different parameter values only slightly affect the result. We used the same values for the two parameters through all the analyses.

Table 1 shows that the ranks of the excluded human seed genes were high. In nine cases, the excluded genes directly interact with another seed and were ranked within the top 1% amongst all the human genes. Of these, two genes (CD36, ICAM1) were ranked in the top five. In 13 out of 14 cases, the excluded genes were ranked within top 3%. The average rank for the excluded human seed genes is 336 (top 2% among all human genes).

**Table 1:** Result of the leave-one-out cross validation for human genes.

Gene symbol	Rank	Top percentage
CD36	1	0.00%
ICAM1	2	0.01%
CR1	14	0.07%
SLC4A1	78	0.44%
NOS2	99	0.55%
GYPC	121	0.67%
HBB	126	0.70%
GYPA	137	0.76%
FCGR2B	159	0.88%
CISH	232	1.29%
TIRAP	277	1.54%
G6PD	378	2.11%
FCGR2A	403	2.25%
TNF	2679	14.9%

We left out one malaria gene from the seed list each time, and determined the rank of this excluded gene using our method. We showed the rank and percentage among all human genes.

We also evaluated the 63 parasite seed genes, and our approach ranked the excluded nodes within the top 5% in 56 out of 62 cases. Table 2 shows the top 10 parasite genes and their ranks in the cross validation. The average rank for the excluded parasite genes is 199 (top 4% among all parasite genes). Less comprehensive data in the parasite genome than in the human genome may contribute to the lower rank (in percentage) of the parasite seed genes. Overall, this analysis demonstrated the utility of the extended random walk to accurately prioritize known malaria genes.

**Table 2:** Top 10 parasite genes in the leave-one-out cross validation.

Gene (ORF name)	Rank	Top percentage
PFD1235W	18	0.37%
PF11 0521	22	0.45%
PF13 0003	29	0.59%
PFD0995C	49	0.99%
PFL1955W	49	0.99%
PFL1950W	50	1.01%
PF07 0050	52	1.05%
PF07 0051	53	1.07%
PFD0630C	53	1.07%
PFD1150C	55	1.11%

### Network-based approach prioritized novel malaria genes other than the seeds

Large amounts of literature have demonstrated strong associations between individual genes and malaria through transcriptional profiling, biological experimenting and genome-wide association studies. These genes include inflammatory responding genes, such as NF*κ*B and CXCL1 [66], parasite protein receptors, such as BSG [67] and PROCR [57], and the genes involving protection against malaria, such as HLA-B [68] and HAVCR1 [69]. We then used all the seeds to generate our ranking for human genes, and examined the rank of 27 malaria genes, which have been validated in previous published studies. Table 3 shows that 12 out of 27 genes were ranked within the top 1%, and a total of 24 genes within the top 5%.

**Table 3:** Rank of other malaria-associated genes from literature.

Gene symbol	Rank	Top percentage
BSG	15	0.08%
IL6	20	0.11%
IFNG	25	0.14%
IL1B	34	0.19%
IL10	38	0.21%
IL8	65	0.36%
IL4	66	0.37%
IL1A	137	0.77%
CD40LG	142	0.79%
HLA-DRB1	145	0.81%
HLA-B	168	0.94%
HAVCR2	179	0.99%
FUT9	183	1.02%
NF*κ*B1	219	1.22%
HBA1	221	1.23%
HBA2	227	1.27%
HLA-DQB1	230	1.28%
HAVCR1	319	1.78%
GNAS	358	1.99%
IFNGR1	380	2.12%
CXCL1	381	2.12%
MBL2	444	2.48%
CCL20	494	2.76%
IL12B	499	2.79%
IFNAR1	954	5.33%
PROCR	1515	8.46%
IL22	2467	13.8%

We also manually examined the top 50 human genes and found interesting predictions. Among them, TLR4 has been suggested to be protective against malaria in certain populations [70,71]. In addition, a recent mouse model experiment [72] has demonstrated that P53 was critical in the liver-stage infection of malaria. Together, the result demonstrated that our gene ranking prioritized novel malaria-associated genes other than the seeds.

### Prioritized genes are enriched by druggable genes

**Figure 2:**
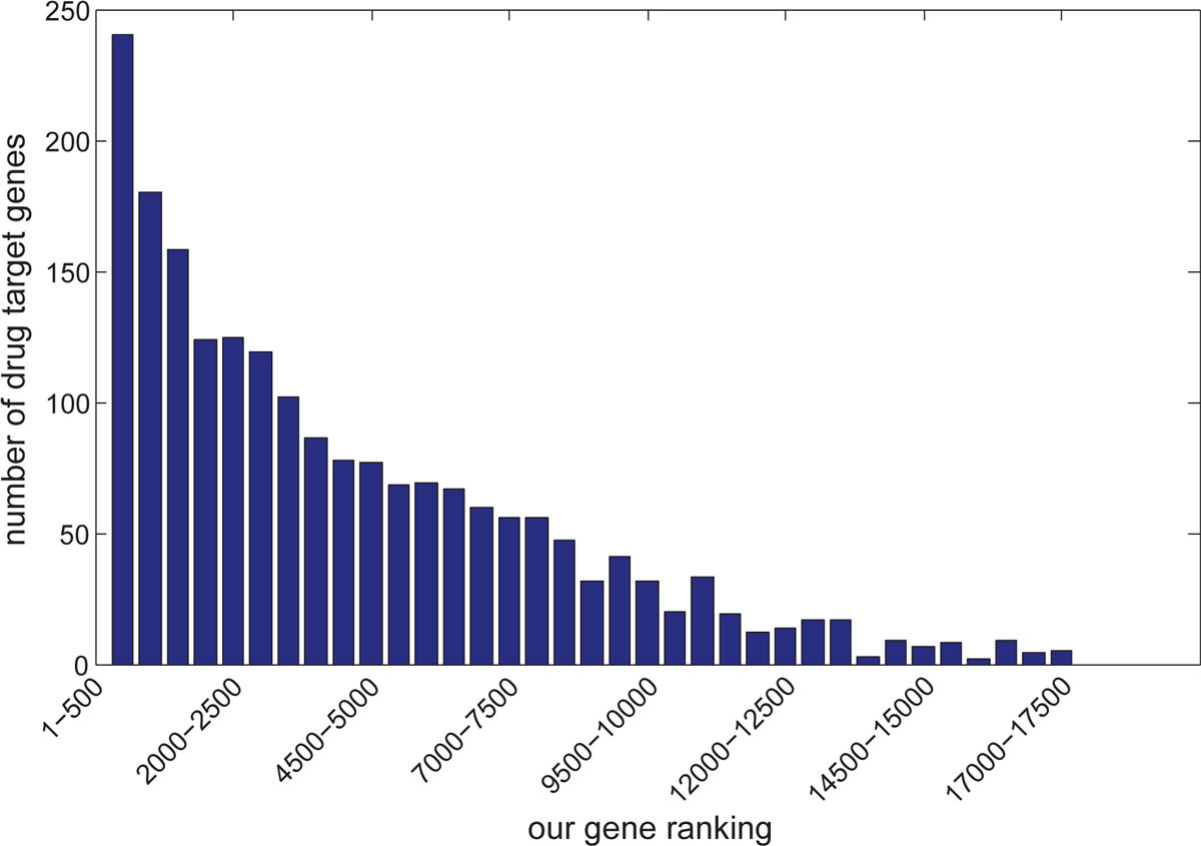
**The count of drug target genes among every 500 genes in our rank from the top to the bottom**.

Figure 2 shows that the top-ranked genes are enriched for drug targets. The top 500 human genes in our ranking have 235 overlaps with the drug targets, which is a 4.3 fold enrichment compared with the average of 100 random rankings (*p <*10^−8^). Among the 235 druggable genes, only 5 have been targeted by FDA-approved anti-malaria drugs, such as chloroquine, proguanil and mefloquine. This result indicated that the top-ranked candidate genes for malaria may provide unique opportunities for malaria drug discovery through novel disease genetics.

### Pathway analysis shows functions of prioritized genes

In order to gain insight into the commonalities underlying predicted malaria candidate genes, we analyzed the pathways associated with top-ranked genes. The top-ranked pathways are associated with different aspects of malaria. For example, malaria parasites actively alter the immune function of B cells and BIOCARTA BLYMPHOCYTE PATHWAY [73]. BIOCARTA LYM PATHWAY is a pathway of lymphocytes adhesion, and plays a central role in binding bacteria, parasites, viruses and tumor cells [74]. Also, BIOCARTA STEM PATHWAY regulates the hematopoiesis and induce hematopoietic activities in the presence of infection [75].

We compared the pathway ranking before and after introducing the parasite genetic network and found nine pathways increased the rank by over 50%. Table 4 lists these pathways and their plausible associations with malaria pathogenesis and protection. Several of these pathways are directly related with the parasite infection and inflammatory responses. REACTOME BASIGIN INTERACTIONS was prioritized through the interaction with the parasite protein PfRh5. Other pathways that were brought up by less than 50% also may have associations with malaria. For example, the rank of the REACTOME HDL MEDIATED LIPID TRANSPORT pathway were improved by 40%. A recent meta-analysis showed that host lipid profile alteration has a link with malaria pathogenesis, though the precise pathway has not been elucidated yet [76].

**Table 4:** Pathways prioritized over 50% in rank.

Pathway	Potential association with malaria
REACTOME PYRUVATE METABOLISM	Pyruvate kinase deficiency protect against malaria [77]
REACTOME BASIGIN INTERACTIONS	Basigin is a receptor essential for erythrocyte invasion by *Plasmodium falciparum *[67]
PID SYNDECAN1 PATHWAY	Induced by parasite infection [78]
REACTOME PYRUVATE METABOLISM AND CITRIC ACID TCA CYCLE	Pyruvate kinase deficiency protect against malaria [77] and citric acid cycle activity involves chloroquine resistance [79]
REACTOME INTEGRIN CELL SURFACE INTERACTIONS	Associated with Plasmodium induced thrombocytopenia [80]
REACTOME CELL SURFACE INTERACTIONS AT THE VASCULAR WALL	Associated with red blood cell adhesion to the endothelial cell and cerebral malaria [81,82]
BIOCARTA VDR PATHWAY	Control cellular nutrient uptake, differentiation, apoptosis, which may be affected by parasites [13,83]
BIOCARTA MONOCYTE PATHWAY	Recruitment and activation of monocytes and macrophages are essential for both protection and pathology in malaria-infected individuals [84]
REACTOME PLATELET ADHESION TO EXPOSED COLLAGEN	Platelet adhesion and aggregation may play important roles in facilitating adhesion of infected red blood cells [85-87]

## Discussion

Malaria is caused by the invasion of deadly parasites into human skin, liver and blood. The parasites trigger the human immune responses, but can manipulate human cells for nutrient uptake and cell growth. Recent studies have shown that host-pathogen protein interactions illuminate the malaria-specific pathways in the human host. With the accumulation of data in both human and parasite genome, systematically analyzing these two interacting genomes may potentially discover new malaria-associated genes, which will pave the way to identify novel anti-malaria drugs.

We developed a data-driven method to infer malaria genes based on random walking on the cross-species genetic networks. We demonstrated that the method can prioritize genes that are both drug targets and associated with malaria. Through comparing the result before and after adding the parasite genetic network into our method, we extracted specific pathways involving human-parasite interactions.

Our approach can be improved with a more comprehensive database of host-pathogen protein interactions. We currently manually curated 36 interactions, mostly from literature, to connect the human and parasite genetic network. Compared with the humanhuman protein interactions, the coverage of humanparasite interaction is much lower and might be biased. As more data are introduced into the method, the global structure of the cross-species genetic network may change, which will affect the result of gene ranking. In the future, we plan to automatically mine the human-parasite interaction from literature and construct a database with better coverage.

Since our approach prioritized a set of druggable genes, which are associated with malaria, one example of subsequent work is to perform drug repositioning through matching the targets of approved drugs to predicted genes. In this way, however, a part of the candidate drugs may target generic inflammatory responses and may not be specific enough to kill the parasites. In addition, malaria is associated with different pathways when human are infected by different parasite species (other than *Plasmodium falciparum*) or different strains [28]. To develop more effective agents against malaria, we need to dissect the genetic basis using more specific data.

## Conclusions

The lack of effective anti-malaria drugs and the poorlyunderstood disease genetics has motivated our study of detecting novel malaria-associated genes from both human and parasite genomes, with the ultimate goal of discovering innovative anti-malaria drugs based on a new genetic understanding of the disease. We developed a data-driven approach to infer malariaassociated genes. Since malaria is caused by the interactions between parasites and human, we constructed a cross-species genetic network to model these interactions, and prioritized relative genes using network analysis. We demonstrated the validity of the method in predicting malaria genes, and showed the potential of the predicted genes in drug discovery. We also extracted pathways from the result of gene ranking, and found these pathways reflect different aspects of malaria pathogenesis.

## Competing interests

The authors declare that they have no competing interests.

## Authors' contributions

RX conceived the study. YC designed the methods, performed the experiments and wrote the manuscript. Both authors have participated study discussion and manuscript preparation.

## Declarations

Publication charges for this article have been funded by the Training grant in Computational Genomic Epidemiology of Cancer (CoGE) (R25 CA094186-06).

This article has been published as part of *BMC Genomics *Volume 16 Supplement 7, 2015: Selected articles from The International Conference on Intelligent Biology and Medicine (ICIBM) 2014: Genomics. The full contents of the supplement are available online at http://www.biomedcentral.com/bmcgenomics/supplements/16/S7.

## References

[B1] World Health Organization. World Malaria Report 2013 http://www.who.int/malaria/publications/world malaria report 2013/en/

[B2] Kar S Kar S Control of malaria Nature Reviews Drug Discovery 2010 16 7 511 512 10.1038/nrd3207 20592740

[B3] Biamonte MA Wanner J Le Roch KG Recent advances in malaria drug discovery Bioorg Med Chem Lett 2013 23 10 2829 2843 10.1016/j.bmcl.2013.03.067 23587422 PMC3762334

[B4] Murray CJ Rosenfeld LC Lim SS Andrews KG Foreman KJ Haring D Global malaria mortality between 1980 and 2010: a systematic analysis The Lancet 2012 379 9814 413 431 10.1016/S0140-6736(12)60034-8 22305225

[B5] Kim Y Schneider K Evolution of drug resistance in malaria parasite populations Nature Education Knowledge 2013 4 8 6

[B6] Dondorp AM Nosten F Yi P Das D Phyo AP Tarning J Lwin KM Ariey F Hanpithakpong W Lee SJ Artemisinin resistance in Plasmodium falciparum malaria New England Journal of Medicine 2009 361 5 455 467 10.1056/NEJMoa0808859 19641202 PMC3495232

[B7] Miller LH Ackerman HC Su Xz Wellems TE Malaria biology and disease pathogenesis: insights for new treatments Nature Med 2013 19 2 156 167 10.1038/nm.3073 23389616 PMC4783790

[B8] Horn D Duraisingh MT Antiparasitic chemotherapy: From genomes to mechanisms Pharmacology and Toxicology 2014 54 1 71 94 10.1146/annurev-pharmtox-011613-135915 PMC4381542 24050701

[B9] Flannery EL Chatterjee AK Winzeler EA Antimalarial drug discovery [MDASH] approaches and progress towards new medicines Nature Reviews Microbiology 2013 11 12 849 862 10.1038/nrmicro3138 24217412 PMC3941073

[B10] Hanson KK Ressurreicao AS Buchholz K Prudencio M Herman-Ornelas JD Rebelo M Torins are potent antimalarials that block replenishment of Plasmodium liver stage parasitophorous vacuole membrane proteins Proceedings of the National Academy of Sciences 2013 110 30 2838 2847 10.1073/pnas.1306097110 PMC3725106 23836641

[B11] Ariey F Witkowski B Amaratunga C Beghain J Langlois AC Khim N A molecular marker of artemisinin-resistant Plasmodium falciparum malaria Nature 2014 505 7481 50 55 24352242 10.1038/nature12876 PMC5007947

[B12] Kwiatkowski DP How malaria has affected the human genome and what human genetics can teach us about malaria The American Journal of Human Genetics 2015 77 2 171 192 10.1086/432519 PMC1224522 16001361

[B13] Menard R Tavares J Cockburn I Markus M Zavala F Amino R Looking under the skin: the first steps in malarial infection and immunity Nature Reviews Microbiology 2013 11 10 701 712 10.1038/nrmicro3111 24037451

[B14] Crompton PD Moebius J Portugal S Waisberg M Hart G Garver LS Malaria immunity in man and mosquito: Insights into unsolved mysteries of a deadly infectious disease* Annu Rev Immunol 2014 32 157 187 10.1146/annurev-immunol-032713-120220 24655294 PMC4075043

[B15] Zheng H Tan Z Xu W Immune evasion strategies of pre-erythrocytic malaria parasites Mediators of Inflammation 2014 2014 10.1155/2014/362605 PMC4033516 24891764

[B16] Engwerda CR Kumar R Mast cells fuel the fire of malaria immunopathology Nature Med 2013 19 6 672 674 10.1038/nm.3227 23744145

[B17] Kaushansky A Metzger P Douglass A Mikolajczak S Lakshmanan V Kain H Kappe SH Malaria parasite liver stages render host hepatocytes susceptible to mitochondria-initiated apoptosis Cell Death Dis 2013 4 8 e762 10.1038/cddis.2013.286 23928701 PMC3763448

[B18] Sicard A Semblat JP Doerig C Hamelin R Moniatte M Dorin-Semblat D Activation of a PAK-MEK signalling pathway in malaria parasite-infected erythrocytes Cell Microbiol 2011 13 6 836 845 10.1111/j.1462-5822.2011.01582.x 21371233 PMC3123749

[B19] Austin LS Kaushansky A Kappe SH Susceptibility to Plasmodium liver stage infection is altered by hepatocyte polyploidy Cellular Microbiol 2014 16 5 784 795 10.1111/cmi.12282 PMC4008336 24612025

[B20] Khor CC Hibberd ML Revealing the molecular signatures of host-pathogen interactions Genome Biol 2011 12 10 229 10.1186/gb-2011-12-10-229 22011345 PMC3333766

[B21] Gardner MJ Hall N Fung E White O Berriman M Hyman RW Genome sequence of the human malaria parasite Plasmodium falciparum Nature 2002 419 6906 498 511 10.1038/nature01097 12368864 PMC3836256

[B22] Elsworth B Matthews K Nie CQ Kalanon M Charnaud SC Sanders PR PTEX is an essential nexus for protein export in malaria parasites Nature 2014 511 7511 587 591 10.1038/nature13555 25043043

[B23] Beck JR Muralidharan V Oksman A Goldberg DE PTEX component HSP101 mediates export of diverse malaria effectors into host erythrocytes Nature 2014 511 7511 592 595 10.1038/nature13574 25043010 PMC4130291

[B24] Bongfen SE Laroque A Berghout J Gros P Genetic and genomic analyses of host-pathogen interactions in malaria Trends Parasitol 2009 25 9 417 422 10.1016/j.pt.2009.05.012 19717339

[B25] Hedrick PW Population genetics of malaria resistance in humans Heredity 2011 107 4 283 304 10.1038/hdy.2011.16 21427751 PMC3182497

[B26] Driss A Hibbert JM Wilson NO Iqbal SA Adamkiewicz TV Stiles JK Genetic polymorphisms linked to susceptibility to malaria Malar J 2011 10 271 10.1186/1475-2875-10-271 21929748 PMC3184115

[B27] Venkatesh S Workman JL Wahlgren M Bejarano MT Malaria: Molecular secrets of a parasite Nature 2013 499 7457 156 157 10.1038/nature12407 23823720

[B28] Wu J Tian L Yu X Pattaradilokrat S Li J Wang M Strain-specific innate immune signaling pathways determine malaria parasitemia dynamics and host mortality Proceedings of the National Academy of Sciences 2014 111 4 E511 E520 10.1073/pnas.1316467111 PMC3910569 24474800

[B29] Janes JH Wang CP Levin-Edens E Vigan-Womas I Guillotte M Melcher M Investigating the host binding signature on the plasmodium falciparum PfEMP1 protein family PLoS Pathog 2011 7 5 e1002032 10.1371/journal.ppat.1002032 21573138 PMC3088720

[B30] Robinson BA Welch TL Smith JD Widespread functional specialization of Plasmodium falciparum erythrocyte membrane protein 1 family members to bind CD36 analysed across a parasite genome Molecular Microbiol 2003 47 5 1265 1278 10.1046/j.1365-2958.2003.03378.x 12603733

[B31] Baruch DI Ma XC Pasloske B Howard RJ Miller LH CD36 peptides that block cytoadherence define the CD36 binding region for Plasmodium falciparum-infected erythrocytes Blood 1999 94 6 2121 2127 10477742

[B32] Baruch DI Gormely JA Ma C Howard RJ Pasloske BL Plasmodium falciparum erythrocyte membrane protein 1 is a parasitized erythrocyte receptor for adherence to CD36, thrombospondin, and intercellular adhesion molecule 1 Proceedings of the National Academy of Sciences 1996 93 8 3497 3502 10.1073/pnas.93.8.3497 PMC39638 8622965

[B33] Bengtsson A Joergensen L Rask TS Olsen RW Andersen MA Turner L A novel domain cassette identifies plasmodium falciparum PFEMP1 proteins binding ICAM-1 and is a target of cross-reactive, adhesion-inhibitory antibodies The Journal of Immunology 2013 190 1 240 249 10.4049/jimmunol.1202578 23209327 PMC3539686

[B34] Smith JD Craig AG Kriek N Hudson-Taylor D Kyes S Fagen T Identification of a Plasmodium falciparum intercellular adhesion molecule-1 binding domain: a parasite adhesion trait implicated in cerebral malaria Proceedings of the National Academy of Sciences 2000 97 4 1766 1771 10.1073/pnas.040545897 PMC26510 10677532

[B35] Brown A Turner L Christoffersen S Andrews KA Szestak T Zhao Y Molecular architecture of a complex between an adhesion protein from the malaria parasite and intracellular adhesion molecule 1 Journal of Biological Chemistry 2013 288 8 5992 6003 10.1074/jbc.M112.416347 23297413 PMC3581401

[B36] Chakravorty S Hughes K Craig A Host response to cytoadherence in Plasmodium falciparum Biochemical Society Transactions 2008 36 Pt 2 221 228 18363564 10.1042/BST0360221

[B37] Medana IM Turner GD Human cerebral malaria and the blood-brain barrier Int J Parasitol 2006 36 5 555 568 10.1016/j.ijpara.2006.02.004 16616145

[B38] Tham WH Wilson DW Lopaticki S Schmidt CQ Tetteh-Quarcoo PB Barlow PN Complement receptor 1 is the host erythrocyte receptor for Plasmodium falciparum PfRh4 invasion ligand. Proceedings of the National Academy of Sciences 2010 107 40 17327 17332 10.1073/pnas.1008151107 PMC2951459 20855594

[B39] Spadafora C Awandare GA Kopydlowski KM Czege J Moch JK Finberg RW Complement receptor 1 is a sialic acid-independent erythrocyte receptor of Plasmodium falciparum PLoS Pathogens 2010 6 6 e1000968 10.1371/journal.ppat.1000968 20585558 PMC2887475

[B40] Barabasi AL Gulbahce N Loscalzo J Network medicine: a network-based approach to human disease Nature Reviews Genetics 2011 12 1 56 68 10.1038/nrg2918 21164525 PMC3140052

[B41] Kohler S Bauer S Horn D Robinson PN Walking the interactome for prioritization of candidate disease genes The American Journal of Human Genetics 2008 82 4 949 958 10.1016/j.ajhg.2008.02.013 PMC2427257 18371930

[B42] Wang X Gulbahce N Yu H Network-based methods for human disease gene prediction Brief Funct Genomics 2011 10 5 280 293 10.1093/bfgp/elr024 21764832

[B43] Aerts S Lambrechts D Maity S Van Loo P Coessens B De Smet F Gene prioritization through genomic data fusion Nature Biotechnol 2006 24 5 537 544 10.1038/nbt1203 16680138

[B44] Lage K Karlberg EO Størling ZM Olason PI Pedersen AG Rigina O A human phenome-interactome network of protein complexes implicated in genetic disorders Nature Biotechnol 2007 25 3 309 316 10.1038/nbt1295 17344885

[B45] Wu X Jiang R Zhang MQ Li S Network-based global inference of human disease genes Mol Syst Biol 2008 4 1 189 18463613 10.1038/msb.2008.27 PMC2424293

[B46] Li Y Patra JC Genome-wide inferring gene-phenotype relationship by walking on the heterogeneous network Bioinformatics 2010 26 9 1219 1224 10.1093/bioinformatics/btq108 20215462

[B47] Wu X Liu Q Jiang R Align human interactome with phenome to identify causative genes and networks underlying disease families Bioinformatics 2009 25 1 98 104 10.1093/bioinformatics/btn593 19010805

[B48] Vanunu O Magger O Ruppin E Shlomi T Sharan R Associating genes and protein complexes with disease via network propagation PLoS Comput Biol 2010 6 1 e1000641 10.1371/journal.pcbi.1000641 20090828 PMC2797085

[B49] Gandhi T Zhong J Mathivanan S Karthick L Chandrika K Mohan SS Analysis of the human protein interactome and comparison with yeast, worm and fly interaction datasets Nature Genetics 2006 38 3 285 293 10.1038/ng1747 16501559

[B50] Franceschini A Szklarczyk D Frankild S Kuhn M Simonovic M Roth A String v9. 1: protein-protein interaction networks, with increased coverage and integration Nucleic Acids Res 2013 41 D1 808 815 10.1093/nar/gks1094 PMC3531103 23203871

[B51] Snel B Lehmann G Bork P Huynen MA String: a web-server to retrieve and display the repeatedly occurring neighbourhood of a gene Nucleic Acids Res 2000 28 18 3442 3444 10.1093/nar/28.18.3442 10982861 PMC110752

[B52] Aurrecoechea C Brestelli J Brunk BP Fischer S Gajria B Gao X EuPathDB: a portal to eukaryotic pathogen databases Nucleic Acids Res 2010 38 Database issue D415 D419 19914931 10.1093/nar/gkp941 PMC2808945

[B53] Gray KA Daugherty LC Gordon SM Seal RL Wright MW Bruford EA Genenames. org: the hgnc resources in 2013 Nucleic Acids Res 2012 Database issue D545 D552 23161694 10.1093/nar/gks1066 PMC3531211

[B54] Aurrecoechea C Brestelli J Brunk BP Dommer J Fischer S Gajria B PlasmoDB: a functional genomic database for malaria parasites Nucleic Acids Res 2009 37 Database issue D539 D543 18957442 10.1093/nar/gkn814 PMC2686598

[B55] Pasternak ND Dzikowski R PfEMP1: An antigen that plays a key role in the pathogenicity and immune evasion of the malaria parasite Plasmodium falciparum Int J Biochem Cell Biol 2009 41 7 1463 1466 10.1016/j.biocel.2008.12.012 19150410

[B56] Flick K Chen Q var genes, PfEMP1 and the human host Mol Biochem Parasitol 2004 134 1 3 9 10.1016/j.molbiopara.2003.09.010 14747137

[B57] Turner L Lavstsen T Berger SS Wang CW Petersen JE Avril M Severe malaria is associated with parasite binding to endothelial protein C receptor Nature 2013 498 7455 502 505 10.1038/nature12216 23739325 PMC3870021

[B58] Park HJ Guariento M Maciejewski M Hauhart R Tham WH Cowman AF Using mutagenesis and structural biology to map the binding site for the Plasmodium falciparum merozoite protein PfRh4 on the human immune adherence receptor Journal of Biological Chemistry 2014 289 1 450 463 10.1074/jbc.M113.520346 24214979 PMC3879568

[B59] Tham WH Wilson DW Reiling L Chen L Beeson JG Cowman AF Antibodies to reticulocyte binding protein-like homologue 4 inhibit invasion of Plasmodium falciparum into human erythrocytes Infect Immun 2009 77 6 2427 2435 10.1128/IAI.00048-09 19307208 PMC2687348

[B60] Williams AR Douglas AD Miura K Illingworth JJ Choudhary P Murungi LM Enhancing blockade of Plasmodium falciparum erythrocyte invasion: assessing combinations of antibodies against PfRH5 and other merozoite antigens PLoS Pathogens 2012 8 11 e1002991 10.1371/journal.ppat.1002991 23144611 PMC3493472

[B61] Douglas AD Williams AR Illingworth JJ Kamuyu G Biswas S Goodman AL The blood-stage malaria antigen PfRH5 is susceptible to vaccine-inducible cross-strain neutralizing antibody Nature Commun 2011 2 601 22186897 10.1038/ncomms1615 PMC3504505

[B62] Douglas AD Baldeviano GC Miura K Wright GJ Draper SJ PfRH5 vaccine efficacy against heterologous strain blood-stage Plasmodium falciparum The Lancet 2014 383 Special issue S43 10.1016/j.chom.2014.11.017 PMC4297294 25590760

[B63] Hopkins AL Groom CR The druggable genome Nat Rev Drug Discov 2002 1 9 727 730 10.1038/nrd892 12209152

[B64] Law V Knox C Djoumbou Y Jewison T Guo AC Liu Y DrugBank 4.0: shedding new light on drug metabolism Nucleic Acids Res 2014 42 D1 1091 1097 10.1093/nar/gkt1068 PMC3965102 24203711

[B65] Subramanian A Tamayo P Mootha VK Mukherjee S Ebert BL Gillette MA Gene set enrichment analysis: a knowledge-based approach for interpreting genome-wide expression profiles Proc Natl Acad Sci U S A 2005 102 43 15545 15550 10.1073/pnas.0506580102 16199517 PMC1239896

[B66] Tripathi AK Sha W Shulaev V Stins MF Sullivan DJ Plasmodium falciparum-infected erythrocytes induce NF-kappaB regulated inflammatory pathways in human cerebral endothelium Blood 2009 114 19 4243 4252 10.1182/blood-2009-06-226415 19713460 PMC2925626

[B67] Crosnier C Bustamante LY Bartholdson SJ Bei AK Theron M Uchikawa M Basigin is a receptor essential for erythrocyte invasion by Plasmodium falciparum Nature 2011 480 7378 534 537 22080952 10.1038/nature10606 PMC3245779

[B68] Hill AV Allsopp CE Kwiatkowski D Anstey NM Twumasi P Rowe PA Common west African HLA antigens are associated with protection from severe malaria. Nature 1991 352 6336 595 600 10.1038/352595a0 1865923

[B69] Nuchnoi P Ohashi J Kimura R Hananantachai H Naka I Krudsood S Significant association between TIM1 promoter polymorphisms and protection against cerebral malaria in Thailand Ann Hum Genet 2008 72 3 327 336 10.1111/j.1469-1809.2007.00424.x 18294362

[B70] Ferwerda B McCall MB Alonso S Giamarellos-Bourboulis EJ Mouktaroudi M Izagirre N TLR4 polymorphisms, infectious diseases, and evolutionary pressure during migration of modern humans Proceedings of the National Academy of Sciences 2007 104 42 16645 16650 10.1073/pnas.0704828104 PMC2034238 17925445

[B71] Sawian CE Lourembam SD Banerjee A Baruah S Polymorphisms and expression of TLR4 and 9 in malaria in two ethnic groups of Assam, northeast India Innate Immunity 2013 19 2 174 183 10.1177/1753425912455675 22948021

[B72] Kaushansky A Ye AS Austin LS Mikolajczak SA Vaughan AM Camargo N Suppression of host p53 is critical for Plasmodium liver-stage infection Cell Reports 2013 3 3 630 637 10.1016/j.celrep.2013.02.010 23478020 PMC3619000

[B73] Scholzen A Sauerwein RW How malaria modulates memory: activation and dysregulation of B cells in Plasmodium infection Trends Parasitol 2013 29 5 252 262 10.1016/j.pt.2013.03.002 23562778

[B74] Vestweber D Blanks JE Mechanisms that regulate the function of the selectins and their ligands Physiological Reviews 1999 79 1 181 213 9922371 10.1152/physrev.1999.79.1.181

[B75] Abdalla S Hematopoiesis in human malaria Blood Cells 1989 16 2-3 401 416 2257320

[B76] Visser BJ Wieten RW Nagel IM Grobusch MP Serum lipids and lipoproteins in malaria-a systematic review and meta-analysis Malaria Journal 2013 12 1 442 10.1186/1475-2875-12-442 24314058 PMC4029227

[B77] Ayi K Min-Oo G Serghides L Crockett M Kirby-Allen M Quirt I Pyruvate kinase deficiency and malaria New England Journal of Medicine 2008 358 17 1805 1810 10.1056/NEJMoa072464 18420493

[B78] Beiting DP Park PW Appleton JA Synthesis of syndecan-1 by skeletal muscle cells is an early response to infection with Trichinella spiralis but is not essential for nurse cell development Infection and Immunity 2006 74 3 1941 1943 10.1128/IAI.74.3.1941-1943.2006 16495570 PMC1418630

[B79] Howells R Maxwell L Citric acid cycle activity and chloroquine resistance in rodent malaria parasites: the role of the reticulocyte Ann Trop Med Parasitol 1973 67 3 285 300 4148616 10.1080/00034983.1973.11686889

[B80] Campos FM Santos ML Kano FS Fontes CJ Lacerda MV Brito CF Carvalho LH Genetic variability in platelet integrin α2β1 density: Possible contributor to Plasmodium vivax-induced severe thrombocytopenia Am J Trop Med Hyg 2013 88 2 325 328 10.4269/ajtmh.2012.12-0297 23249684 PMC3583325

[B81] Mota MM Jarra W Hirst E Patnaik PK Holder AA Plasmodium chabaudi-infected erythrocytes adhere to CD36 and bind to microvascular endothelial cells in an organ-specific way Infection and Immunity 2000 68 7 4135 4144 10.1128/IAI.68.7.4135-4144.2000 10858230 PMC101711

[B82] Nacer A Movila A Baer K Mikolajczak SA Kappe SH Frevert U Neuroimmunological blood brain barrier opening in experimental cerebral malaria PLoS Pathogens 2012 8 10 e1002982 10.1371/journal.ppat.1002982 23133375 PMC3486917

[B83] Zeba AN Sorgho H Rouamba N Zongo I Rouamba J Guiguemd´e RT Major reduction of malaria morbidity with combined vitamin a and zinc supplementation in young children in burkina faso: a randomized double blind trial Nutr J 2008 7 7 7 18237394 10.1186/1475-2891-7-7 PMC2254644

[B84] Chua CLL Brown G Hamilton JA Rogerson S Boeuf P Monocytes and macrophages in malaria: protection or pathology? Trends in Parasitology 2013 29 1 26 34 10.1016/j.pt.2012.10.002 23142189

[B85] Grau GE Mackenzie CD Carr RA Redard M Pizzolato G Allasia C Platelet accumulation in brain microvessels in fatal pediatric cerebral malaria Journal of Infectious Diseases 2003 187 3 461 466 10.1086/367960 12552430

[B86] Pain A Ferguson DJ Kai O Urban BC Lowe B Marsh K Roberts DJ Platelet-mediated clumping of Plasmodium falciparum-infected erythrocytes is a common adhesive phenotype and is associated with severe malaria Proceedings of the National Academy of Sciences 2001 98 4 1805 1810 10.1073/pnas.98.4.1805 PMC29338 11172032

[B87] Wassmer SC Lepolard C Traore B Pouvelle B Gysin J Grau GE Platelets reorient Plasmodium falciparum-infected erythrocyte cytoadhesion to activated endothelial cells Journal of Infectious Diseases 2004 189 2 180 189 10.1086/380761 14722881

